# Case Report: Pulmonary *Mycobacterium avium* complex mimicking recurrent tuberculosis in a patient with suspected pneumoconiosis and destroyed lung diagnosed by bronchoalveolar lavage fluid targeted next-generation sequencing

**DOI:** 10.3389/fmed.2026.1832417

**Published:** 2026-04-22

**Authors:** Chao Xie, Lei Zha, Liqin Zhang

**Affiliations:** Department of Pulmonary and Critical Care Medicine, The First Affiliated Hospital of Wannan Medical College (Yijishan Hospital of Wannan Medical College), Wuhu, Anhui, China

**Keywords:** bronchoalveolar lavage fluid, destroyed lung, *Mycobacterium avium* complex, nontuberculous mycobacteria, suspected pneumoconiosis, targeted next-generation sequencing

## Abstract

A 42-year-old man, a coal miner with a history of pulmonary tuberculosis, dust exposure, and suspected pneumoconiosis, presented with cough, purulent sputum, and intermittent fever for more than 1 month. Routine sputum bacterial and fungal cultures, as well as acid-fast bacilli smears, were persistently negative. Chest computed tomography revealed markedly distorted bilateral lung architecture with patchy and nodular opacities, multiple irregular cavitary lesions, emphysema, pulmonary bullae, and mediastinal lymphadenopathy. To obtain a definitive diagnosis, a bronchoscopy was safely performed under strict asepsis and painless anesthesia. The procedure showed inflammatory bronchial changes and narrowing of the right upper lobe posterior and dorsal segmental bronchi due to external compression. Targeted next-generation sequencing (tNGS) of the bronchoalveolar lavage fluid on the MGI VisionSeq 1000 platform detected *Mycobacterium avium* complex (349 sequence reads, 53.4% relative abundance). The patient’s condition improved rapidly following a targeted multidrug regimen (clarithromycin, rifampicin, ethambutol, and amikacin) combined with supportive pneumoconiosis care. This case highlights the vital role of tNGS in differentiating nontuberculous mycobacterial disease from recurrent tuberculosis in patients with complex structural lung damage, enabling timely and precise intervention.

## Introduction

Pulmonary nontuberculous mycobacterial (NTM) disease commonly occurs in patients with pre-existing structural lung abnormalities, such as chronic obstructive pulmonary disease, prior tuberculosis, or pneumoconiosis, with *Mycobacterium avium* complex (MAC) being one of the most frequent etiological agents (1). In such compromised lungs, cavitary MAC disease can present with clinical and radiological features that closely mimic recurrent pulmonary tuberculosis, creating substantial diagnostic difficulty. Furthermore, traditional microbiological tools present significant limitations; a negative sputum smear or routine culture does not reliably exclude NTM disease due to low sensitivity and lengthy incubation periods (2).

Herein, we report a highly complex case of pulmonary MAC disease in a patient with suspected pneumoconiosis and severe cavitary destroyed lung resulting from previous tuberculosis. We demonstrate how the strategic application of bronchoalveolar lavage fluid (BALF) targeted next-generation sequencing (tNGS) effectively established a definitive diagnosis, differentiated the infection from recurrent tuberculosis, and guided successful multidrug therapy.

## Case description

### Patient information and clinical findings

A 42-year-old Han Chinese man, a coal miner with a documented history of dust exposure, was admitted on January 19, 2026, with a chief complaint of cough, yellow purulent sputum, and intermittent fever (maximum recorded temperature of 37.9 °C) persisting for over 1 month. He had a history of pulmonary tuberculosis and suspected pneumoconiosis. A local hospital had recently suspected recurrent pulmonary tuberculosis but did not initiate standardized treatment. The patient reported no specific family history of genetic diseases and no notable psychosocial abnormalities, though he expressed significant anxiety regarding the unconfirmed tuberculosis diagnosis and sought definitive care at our institution.

On admission, his vital signs were stable: temperature 36.5 °C, pulse 100 beats/min, respiratory rate 20 breaths/min, and blood pressure 119/67 mmHg. Physical examination revealed coarse breath sounds bilaterally, accompanied by dry and wet rales in both lung fields. There was no evidence of digital clubbing, cyanosis, or chest wall deformity.

### Diagnostic assessment

**Figure 1:**
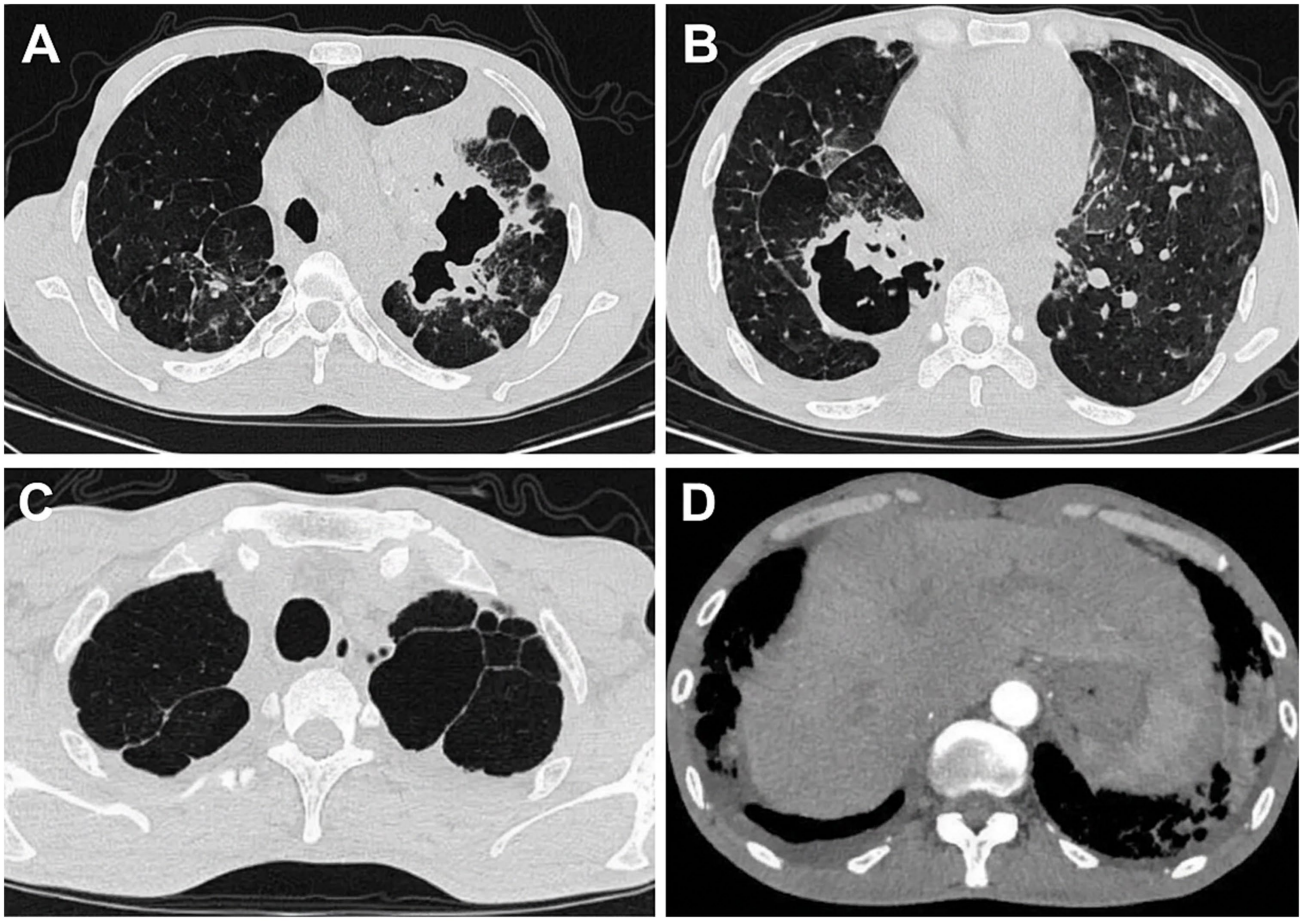
Chest computed tomography findings at admission. **(A)** Left upper lobe cavitary lesion with surrounding patchy and nodular opacities. **(B)** Right lower lobe irregular cavitary lesion with an uneven inner wall and distorted adjacent lung architecture, suggesting partial destroyed lung. **(C)** Emphysematous change and pulmonary bullae. **(D)** Pleural thickening and pleural effusions.

Initial microbiological investigations were non-diagnostic: repeated sputum bacterial cultures showed normal respiratory flora, fungal cultures were negative, and acid-fast bacilli smears were persistently negative. Chest computed tomography (CT) demonstrated severely distorted bilateral lung architecture with multiple patchy and nodular opacities, alongside irregular cavitary lesions, especially prominent in the left upper lobe and right lower lobe. Additional findings included bronchial wall thickening, widespread emphysema, pulmonary bullae, mediastinal lymphadenopathy, and bilateral pleural thickening with effusions (Figure 1). These complex radiological features initially raised a strong clinical suspicion of recurrent tuberculosis complicated by partially destroyed lung.

To secure an etiological diagnosis, a bronchoscopy was scheduled. Under strict asepsis, painless anesthesia was administered intravenously (propofol and fentanyl) followed by the insertion of a laryngeal mask airway. Local anesthesia of the glottis and airways was achieved using lidocaine. Endoscopic examination revealed abundant purulent secretions in the trachea and the lumen of various segments of the left main bronchus, along with mucosal congestion and inflammatory changes bilaterally. The carina was sharp and mobile. The openings of the posterior and dorsal segments of the right upper lobe were narrowed due to external compression. The left upper lobe bronchi were patent, whereas the left lower lobe bronchi were twisted and deformed. No direct signs of neoplasms were observed.

After clearing the secretions, a bronchoalveolar lavage (BAL) was performed in the left lower lobe by instilling 30 mL of sterile normal saline, with a recovery of 15 mL. The BALF was promptly sent for targeted next-generation sequencing (tNGS). The sequencing was performed using the IDseq Focus probe-capture tNGS technology on the MGI tech platform (specifically the VisionSeq 1000 model). The tNGS results identified MAC as the predominant pathogen with 349 specific sequence reads and a relative abundance of 53.4%. *Haemophilus parainfluenzae* was also detected (45 reads, 4.5% relative abundance) but was deemed to be normal oral-pharyngeal flora colonization. Based on the chronic cavitary lung destruction, negative routine sputum studies, and robust tNGS evidence, a definitive diagnosis of pulmonary MAC disease was established.

### Therapeutic intervention

Upon confirmation of MAC infection, a targeted multidrug anti-NTM regimen was initiated. The treatment consisted of clarithromycin (500 mg orally, twice daily), rifampicin (0.45 g orally, once daily), ethambutol (0.75 g orally, once daily), and amikacin (0.4 g in 250 mL normal saline via intravenous infusion, once daily). Concurrently, comprehensive supportive care for pneumoconiosis was administered, including ambroxol and acetylcysteine for expectoration, nebulized budesonide and salbutamol for bronchodilation, and nasal cannula oxygen therapy.

### Follow-up and outcomes

During the initial multi-drug therapy hospitalization period, the patient exhibited excellent tolerability with no reported adverse drug reactions. Regular monitoring confirmed that his liver and kidney functions remained entirely normal. Following this comprehensive treatment strategy, his respiratory symptoms improved significantly, and he remained afebrile. The patient was safely discharged.

During the one-month outpatient follow-up, the patient reported substantial relief from cough and sputum production and remained free of fever. Follow-up laboratory tests indicated that liver and kidney function indices were within normal limits. Although the patient has not yet returned for a follow-up chest CT, his clinical trajectory demonstrates a favorable early response to the targeted intervention.

### Patient perspective

Initially, the patient experienced considerable anxiety when the local hospital suspected recurrent tuberculosis without providing a definitive diagnosis or treatment. Upon presentation to our facility, he was eager for confirmation but expressed apprehension regarding his ability to tolerate the bronchoscopy procedure. However, after thorough preoperative evaluation and reassuring communication with the anesthesiologist, his concerns were alleviated. Following the accurate tNGS diagnosis and the subsequent relief of his symptoms and fever, the patient expressed high satisfaction with his medical care and a significantly improved quality of life upon discharge.

## Discussion

In the present case, the combination of previous tuberculosis, suspected pneumoconiosis (due to occupational dust exposure), and extensive structural lung destruction severely complicated the interpretation of clinical and radiological findings. The CT pattern of multiple cavities and distorted lung architecture initially pointed heavily toward recurrent tuberculosis; however, MAC is notoriously known to present with chronic cavitary disease that perfectly mimics tuberculosis, especially in structurally compromised lungs (3).

Because conventional sputum-based investigations were unrevealing, BALF tNGS provided rapid and clinically actionable etiological evidence. By utilizing the probe-capture tNGS technology on the MGI platform, we were able to obtain a high relative abundance of MAC sequences (53.4%), effectively differentiating a true pathogenic infection from environmental colonization (4).

Concomitant infection of MAC along with other structural lung diseases or carcinomas is a profound topic of interest in the respiratory and infectious disease community. As recently highlighted by Mishra et al. (5), underlying structural distortions provide a fertile niche for MAC colonization and subsequent indolent infection. Particularly in regions with a high epidemiological burden of tuberculosis, clinicians must maintain a high index of suspicion for NTM. Active screening for NTM and the aggressive application of novel diagnostic technologies like tNGS are critical to preventing misdiagnosis and improving the long-term prognosis of patients with underlying structural lung diseases.

A primary strength of this report is demonstrating the successful and safe application of high-risk bronchoscopy and cutting-edge tNGS to identify a fastidious pathogen in a highly complex host. However, the study is limited by its nature as a single case report, and the long-term prognosis of MAC eradication in the setting of severely destroyed pneumoconiotic lungs requires extended follow-up.

In conclusion, this case highlights the crucial role of BALF targeted next-generation sequencing in patients with suspected pneumoconiosis and destroyed lungs when routine microbiological tests are non-diagnostic and pulmonary MAC remains a diagnostic possibility.

## Data Availability

The original contributions presented in the study are included in the article/supplementary material. Further inquiries can be directed to the corresponding author.
